# Symptomatic hypopituitarism revealing primary suprasellar lymphoma

**DOI:** 10.1186/1472-6823-10-19

**Published:** 2010-11-29

**Authors:** Zouhour Fadoukhair, Mounia Amzerin, Nabil Ismaili, Rhizlane Belbaraka, Rachida Latib, Yassir Sbitti, Hind M'rabti, Saber Boutayeb, Mohammed Ichou, Hassan Errihani

**Affiliations:** 1 Department of Medical Oncology, National Institute of Oncology, Rabat, Morocco; 2 Department of Radiology, National Institute of Oncology, Rabat, Morocco; 3 Department of Medical Oncology, Military Instruction Hospital Mohammed V, Rabat, Morocco

## Abstract

**Background:**

The most common cause of hypopituitarism is pituitary adenoma. However, in the case of suprasellar masses different etiologies are possible. We report an unusual case of primary suprasellar lymphoma presented with hypopituitarism.

**Case presentation:**

A 26 year old woman presented with amenorrhea, galactorrhea and neurological disorders. Also, the laboratory work-up revealed partial hypopituitarism. The magnetic resonance imaging of the head showed a suprasellar mass. A presumptive diagnosis of granulomatous processes was made and the patient was given steroid therapy. Repeated brain MRI detected new lesions in the brain with regression of the suprasellar mass. Stereotactic biopsy of the paraventricular lesion revealed the diagnosis of B-cell lymphoma.

**Conclusion:**

This case presentation reports a rare cause of hypopituitarism. Primary suprasellar lymphoma is extremely rare and represented a real diagnostic challenge. Besides, suprasellar masses are varied in aetiology and can present diagnostic problems for a radiologist. Also, because of the increased incidence of PCNSL, lymphoma must be kept in mind in the differential diagnosis of lesions in the suprasellar region.

## Background

The suprasellar region is an anatomically complex area where a number of neoplastic, infectious, inflammatory, developmental and vascular pathologies can occur. The most common etiology of hypopituitarism is the pituitary adenoma, accounting for 10 to 15% of intracranial neoplasms [1]. Differentiation among various etiologies may not always be easy, since many of these lesions may mimic the clinical, endocrinologic and radiologic presentations of pituitary adenomas. The diagnosis of suprasellar lesions involves a multidisciplinary effort. We report an unusual case of primary suprasellar lymphoma presenting as hypopituitarism.

## Case presentation

**Figure 1:**
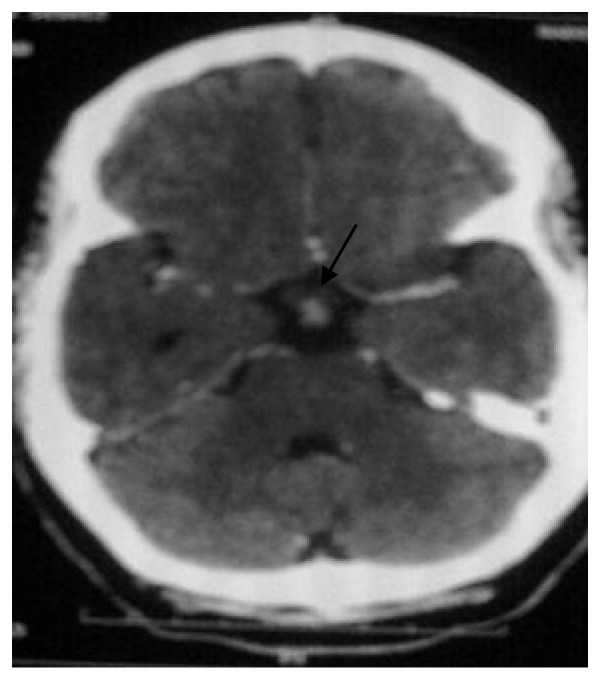
**CT scan of the brain with contrast showing the suprasellar lesion**.

**Figure 2:**
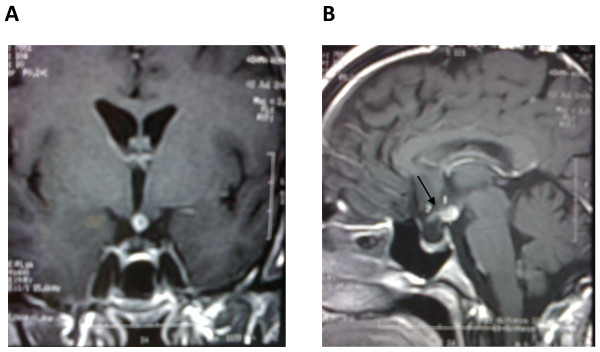
**MRI of the brain coronal (A) and sagittal (B) T1-weighted images with contrast enhancement, performed at the first presentation showing the suprasellar mass(1) with thickening of pituitary stalk (2)**.

A 26 year-old-woman, with a history of infertility for five years (treated by ovulation induction medications), was admitted to the hospital for evaluation of amenorrhea, galactorrhea and neurological disorders. She was well until 6 months before her admission in the neurology department of the University Hospital of Rabat. She developed weakness, headaches associated with nausea and vomiting, shaking chills, night sweats, an 8-pound weight loss and diplopia. Computed tomography scan (CT scan) of the brain showed a hyperdense mass in the suprasellar region (Figure 1). Magnetic resonance imaging (MRI) of the brain (T1 and T2 weighted with contrast) revealed an enhancing suprasellar mass (9 × 6 mm) and thickening of pituitary stalk (Figure 2).

Laboratory findings showed: microcytic anemia (10 g/dl), hyponatremia (130 mEq/L), elevated erythrocyte sedimentation rate (40 mm in first hour) with an increased alpha-2-gobulin in serum protein electrophoresis. An endocrinological evaluation revealed low levels of follicle stimulating hormone (2.5 IU/L; normal ranges in the laboratory 5-20 IU/L) and luteinizing hormone (0.5 IU/L; normal ranges in the laboratory 10-70 IU/L), serum prolactin level was greatly increased (145 ng/ml; normal ranges in the laboratory 2.8-29.2 ng/ml) without dysfunctions of Antidiuretic Hormone. She underwent an extensive biological work-up evaluation including: thyroid function tests, skin testing for tuberculosis; serological tests for HIV, panel hepatitis and syphilis; lumbar puncture and salivary gland biopsy; all were unrevealing. Chest x-ray and abdominal echography were normal.

**Figure 3:**
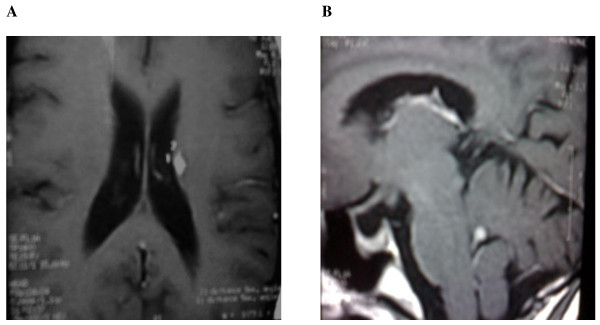
**Four months later after steroid treatment, MRI of the brain showing new process developed in the CNS**.

**Figure 4:**
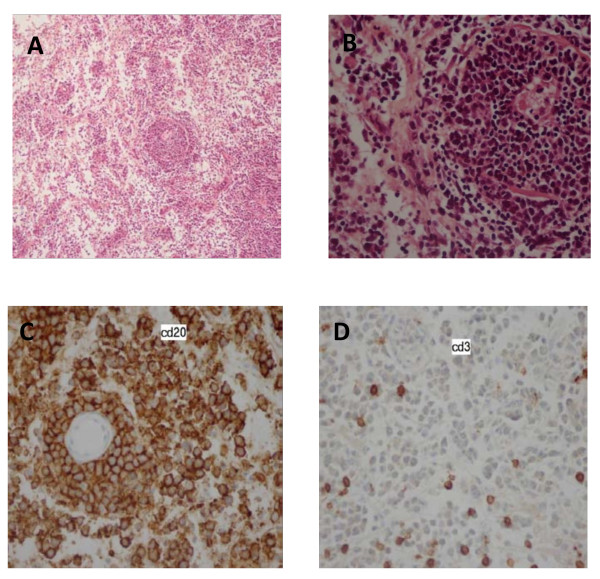
**The histological examination confirmed the diagnosis of pituitary lymphoma**. Figures A and B shows infiltrative large-sized lymphocytes with occasional mitotic. The immunohistocemical tests were positive for the B-cell CD20 marker (C) and negative for the T-cell marker CD3 (D).

Initially, the diagnosis of granulomatous processes was suggested, on the basis of the patient's presentation and imaging findings. Then the patient was given prednisolone 60 mg/day with clinical improvement. However, subsequent head MRI detected new lesions of the brain in contrast with regression of the first mass (Figure 3). She underwent stereotactic biopsy of the paraventricular lesion. Histological examination revealed infiltrative large-sized lymphocytes with occasional mitotic figures. The immunohistochemical tests confirmed the diagnosis of large B-cell lymphoma (the B-cell marker CD20 was positive and the CD3 T-cell marker was negative) (Figure 4). CT scan of the chest, abdomen, and pelvis were negative.

The patient died of hydrocephalus before the initiation of treatment two weeks after the diagnosis.

## Discussion

We described an unusual presentation of brain lymphoma revealed by hypopituitarism. Our patient presented a partial hypopituitarism with a suprasellar mass in brain MRI. Pituitary adenoma is the most common cause of hypopituitarism [1]. Suprasellar masses are varied in aetiology; craniopharyngioma, Rathke cleft cyst, germ cell tumour and granulomatous processes are others common differential diagnosis [1]. However, primary lymphoma of the suprasellar region is extremely rare and represents a diagnostic challenge. Besides, suprasellar masses can present diagnostic problems for a radiologist. MRI findings for pituitary adenomas with suprasellar extension typically have a "figure of eight" or "snowman" appearance and are strongly enhanced. Craniopharyngioma have a heterogeneous appearance with solid and cystic elements tumour in the suprasellar region. Rathke cleft cysts usually have the following imaging features: a sellar epicentre, smooth contour, lack of calcification, lack of internal enhancement, and a homogeneous signal intensity within the lesion. The characteristic features of granulomatous diseases are thickening of the pituitary stalk and absence of a normal, bright posterior pituitary signal. Imaging of germ cell tumour shows generally an infiltrative, solid, homogeneous mass in the midline with intense contrast enhancement [1]. Nevertheless, there are no significant distinctive radiologic characteristics of suprasellar lymphoma. More often, the MRI findings in primary CNS lymphoma are mass lesions that are iso- to hypointense on T1- and T2-weighted images and tend to have strong homogenous enhancement following contrast administration in immunocompotent patients, as in the present cases [1]. The absence of T2 prolongation results from the dense cellularity and high nucleus-to-cytoplasm ratio of lymphoma, and it may help in the differentiation of primary CNS lymphoma from other brain tumours [1-3].

To our knowledge, only nineteen cases of primary lymphoma involving the hypothalamic-pituitary region have been previously reported (Table 1) [2-20]. In the present case the diagnosis of granulomatous processes has been made on the basis of patient presentation and imaging findings. The treatment was subsequently initiated with steroid therapy and showed clinical improvement and suprasellar mass regression. Though, subsequent head MRI detected new lesions in the cerebellum and periventricular region (Figure 3). Then, the diagnosis of primary central nervous system lymphoma (PCNSL) was strongly support according to neuroimaging work-up, because the vast majority of PCNS tumors arise in the deep hemispheric periventricular white matter, the corpus callosum, cerebellum, orbits, and cranial nerves [21].

**Table 1:** Clinical characteristics of 20 patients with primary sellar and suprasellar lymphoma.

Authors and Year (Ref)	Age Sex	Clinical Presentation	Radiological findings	Origin
**Singh et al., 1993 **[2]	28, M	HA, visual loss, facial numbness	CT: lesion in suprasellar, and right parasellar region extending into the sphenoid sinus	B-Cell
**Samuels et al., 1994 **[3]	49, M	HA, nystagmus, decreased libido, hypopit, DI, PRL	MRI: enhancement of a suprasellar mass with infiltration of contiguous structures	B-Cell
**Gottfredsson et al., 1996 **[4]	48, M	HA, nausea/vomiting, meningismus, fever, diplopia	CT: 9 mm enhancing mass in the pituitary region	B-Cell
**Shaw et al., 1997 **[5]	73, F	HA, fatigue, diplopia, polyuria, hypopit, DI, PRL	MRI: mass filling sphenoid sinus on the right, contiguous with pituitary	B-Cell
**Li et al., 1998 **[6]	77, M	Weakness, hypopit	-	B-Cell
**Sakakibara et al., 1998 **[7]	53, M	HA, diplopia	-	T-Cell
**Freda et al., 1999 **[8]	48, M	HA, diplopia	MRI: mass involving suprasella, sphenoid sinus, and cavernous sinus	B-Cell
**Kuhn et al., 1999 **[9]	67, F	Diplopia, hypopit	CT/MRI: large intrasellar, suprasellar, and right parasellar lesion invading the right cavernous sinus	T-Cell
**Au et al., 2000 **[10]	83, M	HA, visual loss, hypopit, DI	MRI: isointense bilobed tumor of pituitary fossa with central hemorrhagic area. Compressed hypothalamus and optic chiasm	B-Cell
**Mathiasen et al., 2000 **[11]	65, M	Weakness, decreased libido, hypopit, PRL	MRI: homogenously enhancing sellar and suprasellar mass	B-Cell
**Singh et al**., **2000 **[12]	44, M	HA, visual loss, decreased libido	MRI: enhancing mass involving sella, and parasellar regions with infiltration of the cavernous sinus.	B-Cell
**Spina et al., 2000 **[13]	52, F	HA, diplopia, hypopit, DI	-	T-Cell
**Landman et al., 2001 **[14]	86, F	Fever, chills, weight loss, hypopit, DI	MRI: mass in pituitary fossa extending into suprasellar cistern. Isointense on T1, enhancing postgadolinium	B-Cell
**Silfen et al., 2001 **[15]	15, F	Polyuria, polydipsia, weight loss, hypopit, DI	MRI: 9 mm enhancing lesion in the pituitary stalk	B-Cell
**Kaufmann et al., 2002 **[16]	74, M	Visual loss, mental status change, hypopit	MRI: sellar and suprasellar masse and extension into the cavernous and sphenoid sinuses	B-cell
**Katz et al., 2003 **[17]	64, F	nausea/vomiting, diarrhea, hypopit, DI	MRI: minimal enlargement of the pituitary region	B-Cell
**Huang et al., 2005 **[18]	47, M	Fever, chills, decreased libido, hypopit	CT: homogeneous enhanced pituitary region mass (22 × 14 mm)	T-Cell
**J.K Liu et al., 2007 **[19]	26, M	HA, diplopia, hypopit	MRI: enhancing sellar mass with suprasellar extension compressing the optic chiasm.	NK/T-Cell
**Krypciak et al., 2010 **[20]	78, F	Weakness, weight loss, nystagmus, hypopit, PRL	CT: 10 mm enhanced pituitary mass	B-Cell
**Present study**	26, F	HA, weight loss, amenorrhea, diplopia, nystagmus, hypopit, PRL	MRI: enhancing suprasellar mass	B-Cell

In the series of patients reported (Table 1), only in one case was the presumptive diagnosis lymphoma of the brain. In the large majority of cases presumptive diagnosis was pituitary adenoma. Confirmation of diagnosis was most frequently obtained with surgery [2-20].

In our review of 20 cases of sellar and suprasellar lymphoma including our patient, we found that the mean age of patients was 55.5 years (range 26 - 86 years). 60% of the patients were male and 40% were female. By contrast, our patient was a 26 year-old-woman.

The most common presentation was hypopituitarism (75%), followed by headache (55%), diplopia (40%), diabetes insipidus (31%) and hyperprolactinemia (25%). Histologically, similar to PCNSL, most lymphomas of the suprasellar region are B-cell non-Hodgkin lymphoma. The MRI of the head demonstrated enhancing parasellar masses with diffuse enlargement of the pituitary gland (95%), suprasellar lesion (45%), cavernous sinus extension (35%), and stalk thickening (20%). Indeed, the present case had hypopituitarism at presentation and brain MRI shows suprasellar lesion and stalk thickening.

Nevertheless, primary central nervous system lymphoma (PCNSL) represents approximately less than 2% of primary brain tumors. Its incidence has increased over the last 30 years [19]. So far, despite recent therapeutic advances, PCNSL exhibit one of the worst prognoses among all non-Hodgkin lymphomas (median survival < 6 months) [22]. Our patient died two weeks after diagnosis of hydrocephalus.

For a long time, radiotherapy (RT) has been the standard treatment, producing a response rate of 60-65% and a notable neurological improvement in most cases of PCNSL. However, relapse usually occurred within a few months after RT. Although the introduction of systemic chemotherapy based on CHOP (cyclophosphamide, doxorubicine, vincristin and prednisone) regimen and high-dose methotrexate followed by radiation therapy has consistently improved survival, the prognosis of PCNSL still dismal, with high rates of local relapse and consequent death. About half of the patients reported received chemotherapy, only in three cases without cranial radiation [22]. Regimens used were different and were in the most cases extrapolated from the protocol used in PCNSL. Despite the increasing number of studies published since a decade on PCNSL and recent therapeutic advances, several questions still remain unanswered about the optimal management of these tumors.

This case presentation reports a rare cause of hypopituitarism. Primary suprasellar lymphoma is extremely rare and represented a real diagnostic challenge. Suprasellar masses are varied in aetiology and can present diagnostic problems for a radiologist. But the differential diagnosis can be narrowed down by taking into account the patient's age, clinical presentation, and imaging appearances. Also, because of the increased incidence of PCNSL, lymphoma must be kept in mind in the differential diagnosis of lesions in the suprasellar region.

## Conclusion

This case presentation reported a rare cause of hypopituitarism. Suprasellar region may be affected by a wide variety of tumors. Nevertheless, primary suprasellar lymphoma is extremely rare and represented a real diagnostic challenge. This case demonstrates that lymphoma must be kept in mind in the differential diagnosis of lesions in the suprasellar region because of the increased incidence of PCNSL.

## Competing interests

The authors declare that they have no competing interests.

## Authors' contributions

ZF performed literature review, the composition of this case report and manuscript writing.

NI-MA-RB conception and design collection and assembly of data.

RL performed radiologic interpretation.

YS-SB-HM-MI- HE analyses and interpretation of data, manuscript writing.

All authors read and approved the manuscript.

## Pre-publication history

The pre-publication history for this paper can be accessed here:

http://www.biomedcentral.com/1472-6823/10/19/prepub
